# Multilevel Correlates of Non-Adherence in Kidney Transplant Patients Benefitting from Full Cost Coverage for Immunosuppressives: A Cross-Sectional Study

**DOI:** 10.1371/journal.pone.0138869

**Published:** 2015-11-30

**Authors:** Elisa Oliveira Marsicano, Neimar Silva Fernandes, Fernando Antônio Basile Colugnati, Natalia Maria Silva Fernandes, Sabina De Geest, Helady Sanders-Pinheiro

**Affiliations:** 1 Renal Transplantation Unit, Division of Nephrology, School Hospital of Federal University of Juiz de Fora, Minas Gerais, Brazil; 2 Núcleo Interdisciplinar de Estudos e Pesquisas em Nefrologia (NIEPEN), Juiz de Fora, Minas Gerais, Brazil; 3 Centre for Public Policy and Education Evaluation (CAED), Federal University of Juiz de Fora, Minas Gerais, Brazil; 4 Institute of Nursing Science, University of Basel, Basel, Switzerland; 5 Department of Public Health and Primary Care, Faculty of Medicine, KU-Leuven, Belgium; University of Toledo, UNITED STATES

## Abstract

**Background:**

Adherence is the result of the interaction of the macro, meso, micro, and patient level factors. The macro level includes full coverage of immunosuppressive medications as is the case in Brazil. We studied the correlates of immunosuppressive non-adherence in post kidney transplant patients in the Brazilian health care system.

**Methods:**

Using a cross-sectional design, adherence to immunosuppressives was assessed in a sample of 100 kidney transplant patients using a composite non-adherence score consisting of three methods (self-report [i.e., The Basel Adherence Scale for Assessment of Immunossupressives–BAASIS], collateral report, and immunosuppressive blood levels). Multilevel correlations of non-adherence were assessed (macro, meso, micro and patient level). Univariate and multivariate logistic regression was applied to assess the correlates of non-adherence.

**Results:**

Our sample consisted primarily of male (65%), Caucasians (72%) with a mean age of 45.0 ± 13.5 years old, who received grafts from a living donor (89%), with a mean time after transplantation of 72.3 ± 44.4 months. Prevalence of non-adherence was 51%. Family income higher than five reference wages (21.6 vs. 4%; OR 6.46 [1.35–30.89], p = 0.009; patient level), and having access to private health insurance (35.3% vs. 18.4%; OR 2.42 [0.96–6.10], p = 0.04; meso level) were associated with non-adherence in univariate analysis. Only the higher family income variable was retained in the multiple logistic regression model (OR 5.0; IC: 1.01–25.14; p = 0.04).

**Conclusions:**

Higher family income was the only factor that was associated with immunosuppressive non-adherence. In Brazil, lower income recipients benefit from better access to care and coverage of health care costs after transplantation. This is supposed to result in a better immunosuppressive adherence compared to high-income patients who have experienced these benefits continuously.

## Introduction

Kidney transplant (KTx) is considered the best therapeutic option for patients with chronic kidney disease (CKD), both from a clinical and economic point of view [1]. In terms of preserving long-term graft function and reducing the risk of complications, KTx recipients have to adhere to a lifelong immunosuppressive regimen since non-adherence (NA) is associated with poor clinical and economic outcomes [2,3,4,5]. A transplant consensus conference defined NA to immunosuppressives (IS) as “deviation from the prescribed medication regimen sufficient to adversely influence the regimen’s intended effect” [6]. NA is a major driver of graft outcomes. An estimated 15% to 60% of late acute rejections and 5% to 36% of graft losses are associated with NA in KTx patients. Non-adherent patients have a 7 fold increased risk of graft loss [7,8,9].

WHO proposes that NA to treatments is a result of a complex, multidimensional, and multilevel interaction between socioeconomic factors, condition, treatment and patient related factors as well as health care team and health system characteristics [5]. Yet, whereas most of the studies have focused on patient characteristics, socioeconomic conditions and treatment related factors, but health care team, and system related factors are still not well understood [9,10]. It is increasingly recognized that the lack of variability explained in adherence research is also due to the absence of analyses of the healthcare systems perspective. It is therefore important to use an ecological perspective for studying NA including the different levels of the health care system such as patient, health care provider (micro), healthcare organization (meso) and health care policy (macro) [11,12].

*Patient-level factors* include, for instance, individual characteristics, self-efficacy, and attitudes. *Micro-level factors* refer to the quality of interpersonal relationships with health care professionals and social support. *Meso-level factors* encompass the characteristics of the health care organization where the patient is being treated (i.e., regularity of follow up). *Macro-level factors* refer to the characteristics of the health care system where the patient lives and includes local, state, and national laws and policies related to health such as insurance coverage and regulations on reimbursement for medication [12,13].

Understanding risk factors for NA provides a basis for targeting modifiable factors through preventive and restorative interventions [5,14]. Given the interplay between the different levels of the system, studying multilevel correlates of NA in a specific health care setting is highly relevant [12].

Brazil is specific case in transplantation, both in volume and in overall health policy framework for transplantation. The number of transplants in Brazil has progressively increased in the last ten years. Although Brazil is ranked 2nd in the absolute number of kidney transplants performed worldwide, the rate of 28.5 kidneys transplants pmp is still far below the country´s requirement [15,16]. Nevertheless, the assurance of a favorable clinical outcome through high quality clinical management remains a controversial issue [15]. All Brazilian citizens are supported by The Unified Health System (SUS), a public health system that is oriented towards the collective interest. In the field of kidney transplantation, the SUS is supposed to cover outpatient follow-up, laboratory exams, medicines, and hospitalizations, all under specific legislation. The special drugs, such as immunosuppressants, are centrally purchased by the government for further local distribution [17]. Otherwise, the Brazilian health system generally provides a less efficient access to exams and specialist consultations, and these limitations also frequently affect the transplant patients. Although the Brazilian public health system guarantees the delivery of all the prescribed immunosuppressives, this efficiency does not uniformly apply to the other aspects of the treatment. For example, there is often difficulty in scheduling specialist consultations or delay in the prompt availability of laboratory exams as well a suboptimal structural condition of hospitals. As consequence, the portion of the population with less economic limitations often chooses to hire private health insurance to complement their treatment [18].

Given the inherent challenges of the Brazilian Health Care System in view of transplantation and their potential specific impact on NA levels, and given the limited evidence in the view of the magnitude and risk factors of NA to immunosuppressives in Brazil, this study aims to determine the prevalence of NA to immunossuppresives in a Brazilian KTx population, applying standardized methods and to explore multilevel risk factors of NA.

## Materials and Methods

The local Ethics in Research Committee (Comitê de Ética do Hospital Universitário da Universidade Federal de Juiz de Fora) approved this study (approval number—0068/2010).

### Design

We conducted a cross sectional study in a single kidney transplant center in Brazil between May to December 2010.

### Sample and Setting

We evaluated a convenience sample of 100 KTx patients followed up in the outpatient clinic of the Núcleo Interdisciplinar de Estudos, Pesquisas e Tratamento em Nefrologia of the Federal University of Juiz de Fora, in Brazil. Patients were included if they were at least 18 years old, more than a year post-transplant, and provided written informed consent. Exclusion criterion was retransplant.

### Variables and Measurements

We retrieved demographic and specific clinical variables from the medical files.

#### NA to immunosuppressive drugs

In the absence of a gold standard for the assessment of NA, triangulation of different diagnostic methods is favored [19,20]. A validation study testing the diagnostic value of a self-report, collateral report and blood assay compared to electronic monitoring demonstrated that a combination of these methods showed the highest sensitivity [21]. We therefore assessed NA to immunosuppressives using self-report, collateral report and assay.

Self-report: We used The Basel Assessment of Adherence Scale for Immunosuppressives (BAASIS), of which the Brazilian Portuguese version was recently validated by our group (reliability Cronbach alpha 0.7) [22]. The four-items of the BAASIS assess the following dimensions of medication NA over the past four weeks: taking adherence (omission of single doses), drug holidays (omission of successive doses), timing adherence (timing deviations of >2 hours), and dose reductions. Responses are given on a six-point scale: never (0), once per month (1), every second week (2), every week (3), more than once per week (4), and every day (5). NA is defined as any deviation from the dosing schedule [20].

Collateral report was provided by the nurse and the medical assistant directly responsible for the follow-up care of the KTx recipients. They categorized patients as good, fair, or poor adherers. NA was defined as a fair or poor upon evaluation by one or both clinicians [21].

Assay: Patients´ adherence was also measured trough blood levels of the immunosuppressives drugs. Based on our service’s adopted guidelines, which consists of a therapeutic range of 100–150 ng/ml for cyclosporine, 5–10 ng/ml for sirolimus and tacrolimus, blood levels were analyzed in five time points: at the moment of the BAASIS interview and at the four prior outpatient clinic appointments. Patients with blood levels within the therapeutic range used by our center were categorized as adherent and those with levels below the accepted values were considered as non-adherent. The study staff performed clinical evaluations of patients with deviating values before they were categorized as non-adherent. Interpretation was based on the patient’s medical conditions as well as their medication blood levels [10].

Composite adherence score: The findings from the self-report, collateral report, and blood assay were combined to calculate a composite adherence score [18]. Overall NA was defined as a diagnosis of NA as assessed for either one of the three methods [21].

#### Correlates of NA

Multilevel correlates of NA included were following. At *Patient-level*, factors included were Socio-economic factors: age (years and younger than 25 years old), gender, race (self-defined; white or other), highest level of education (primary, secondary, and higher education), employment status (yes or no), marital status (married/steady partner or not), family income (per month, categorized in less than or equal to/more than five reference wages–US$ 1,100.00) [23], current smoking and alcohol intake (yes or no); Condition-related factors: time on dialysis (months), the treatment modality previous to transplant (peritoneal, hemodialysis), preemptive transplant (yes or no), donor type (deceased, living-related, or living-unrelated donor graft), and post-transplant time (months); and Therapy-related factors: number of prescribed medications and the daily number of dosing times of the immunosuppressives. At the *Micro-level*, the patients evaluated the quality/satisfaction of their relationship with their health care providers (physician and nurse) by rating these as good, fair, or poor [24]. At the *Meso-level*, city of origin (same or different from transplant´s center) and access to the center (measured by the distance to transplant center (in miles; if more than 62 miles), were evaluated. At the *Macro-level*, private health insurance coverage (yes or no) was measured [12].

### Data Collection

**Fig 1:**
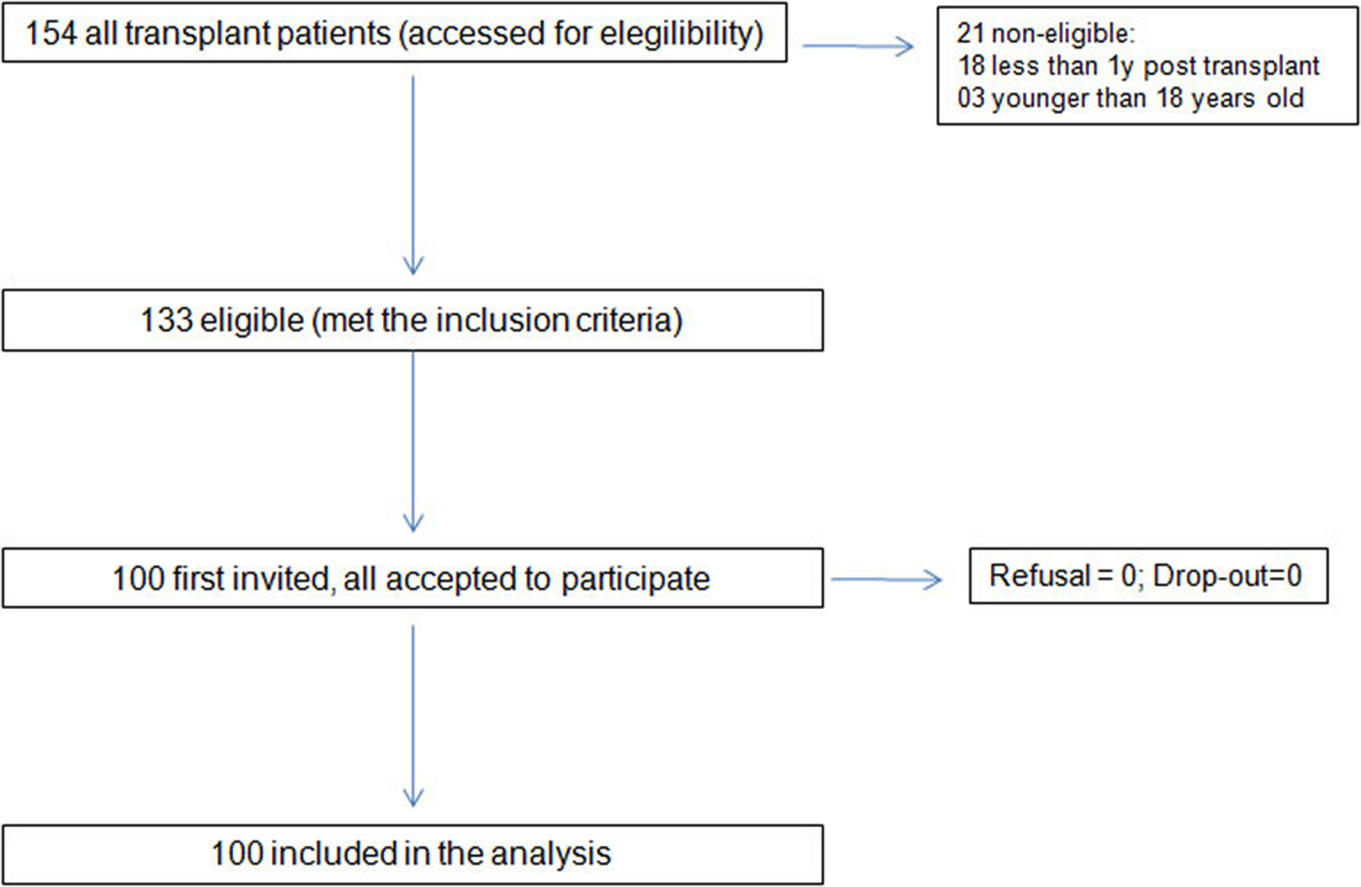
Data collection process involving the screening and determination of eligibility of study participants according to the inclusion criteria.

All patients attending routine visits between 1 May to 1 December 2010, who fulfilled the study inclusion criteria and did not have the exclusion criterion, were invited to participate in the study. They received written and oral information before signing the written informed consent form. Trained nurse interviewers who were part of transplantation team administered the BAASIS. Both the renal nurse and the physician treating the patient filled out the collateral report form. The blood assay was collected from the medical files. Correlates were assessed by interviewers or were collected from the medical files (Fig 1).

### Statistical Procedures

Baseline characteristics were described as frequencies or as mean ± standard deviation and median/interquartile ranges as appropriate, after checking the level of measurement and distribution of variables using the Kolmogorov-Smirnov and Shapiro-Wilk tests. The T-Test, Chi-square, Mann-Whitney, or Fisher´s tests were used to compare adherent with non-adherent patients according to the assessed correlates.

Multivariate Logistic Regression analysis was performed to assess the association between the variables with NA as the outcome. We only included the variables (family income; distance to transplant center; and private health insurance) with a p value of <0.20 in the univariate analysis in the model. Statistical analysis was performed using the SPSS software v.19.0 (Chicago, IL, USA). A p value <0.05 was considered significant. All collected data is summarized in S1 Appendix.

## Results

### Sample Characteristics

From a total of 154 patients, 133 were determined to be eligible for the study. We included the first 100 who accepted the invitation to participate (Fig 1). Given the small sample size, statistical power is expected to be low. If we assume a 0.05 level for type I error, estimated power is 75% for Odds Ratios (OR) > 4 (the effect-size). However, given OR > 3 and assuming a significance level of 10%, statistical power is at least 64%, increasing to 76% if type I error is 20%. Table 1 shows the characteristics of the study participants. Majority of the patients were male (65%), Caucasian (72%), with a mean age of 45 ± 13.5 years. Most received a living donor graft (89%) with a mean post-transplant time of 72.3 ± 42.4 months and a mean creatinine of 1.56 ± 0.56 mg/dL. The majority had finished at least secondary school. Only a third lived in the city of the transplant center, with 37% coming from a neighboring state. Demographic and clinical characteristics (age, sex, time on dialysis, type of donor) of the 21 non-included patients did not differ from the study participants (Fig 1).

**Table 1:** Demographic And Clinical Characteristics Of The Study Participants (N = 100).

Variable	%/N–mean±SD/median (IQR)
**Age (years)**	45.0 ± 13.5
**Male gender**	65% (65/100)
**White race**	72% (72/100)
**Pre-transplant treatment modality**	
Hemodialysis	79% (79/100)
Peritoneal	8% (8/100)
Pre-dialysis	13% (13/100)
**Type of donor**	
Living related	90% (90/100)
Living unrelated	7% (7/100)
Deceased	3% (3/100)
**Post-transplant time (months)**	72.3 ± 42.4
**Time on dialysis (months)**	20.0 (12–36)
**Highest education level**	
Primary School	45% (45/100)
Secondary School	25% (25/100)
Higher Education	30% (30/100)
**City of origin**	
Transplant center	30% (30/100)
Other cities in the same state	43% (43/100)
Other states	27% (26/100)
**Absolute distance from transplant center (miles)**	45.5 (0–96)

SD–standard deviation; IQR–interquartile range. Normality test checked by Kolmogorov-Smirnov and Shapiro-Wilk tests.

### Prevalence of NA

The composite adherence score showed that 51% of patients were non-adherent. Table 2 lists the prevalence per assessment method: self-report (34%), collateral report (48%) assay (7%) and the combination of these three methods.

**Table 2:** Prevalence Of NA To Immunosuppressives According To Measurement Method.

Method	% NA
BAASIS (self-report)	34%
Collateral report	30%
Blood assay	7%
BAASIS+ Collateral report + Blood assay	51%

Non-adherence definitions by each method

BAASIS–self-report; any deviation from taking, timing, reducing or drug holidays dimensions of medication non-adherence.

Collateral report–fair or poor adherence to by health care professionals judgment.

Blood assay–at least one of the last five of immunesuppressive levels below the accepted reference.

#### Multilevel correlates of NA

Table 3 presents the results of the univariate analysis comparing the non-adherent to adherent groups. At patient level, a better family income (i.e., more than five Brazilian reference wages) was the only parameter associated with NA (21.6 vs. 4.1%, p = 0.009). Otherwise, having private insurance coverage was not associated with adherence (35.3 vs. 18.4, p = 0.04). Table 3.

**Table 3:** Comparison Of The Multilevel Correlates Between Adherent And Non-Adherent Patients Using The Composite Score.

Variable	Total N = 100	NA = 51	Adherent N = 49	p-value	OR (Lower ─ Upper)
**Patient level**					
**Socio-economic**					
Age (years)	45.0 ± 13.5	45.7±13.7	44.1±13.3	0.50	
Younger than 25 years	7.0%	9.8% (5)	4.1% (2)	0.25	2.56(0.47**─**13.88)
Male	65.0%	60.8%(31)	69.4%(34)	0.36	0.68(0.29**─**1.56)
White	72.0%	76.5%(39)	67.3%(33)	0.31	1.57(0.65**─**3.80)
Married	62.0%	56.9%(29)	67.3%(33)	0.28	0.63(0.28**─**1.44)
Employed	34.0%	41,2%(14)	58,8%(20)	0.26	0.62(0.26**─**1.43)
Education level					
Primary and Secondary School	79.0%	72.5%(37)	85.7%(42)	0.40	2.27(0.82**─**6.22)
Higher Education	21.0%	27.5%(14)	14.3% (7)		
Family income*					
≤ Five reference wages	87.0%	78.4%(40)	95.9%(47)	0.009	6.45(1.35**─**30.89)
> Five reference wages	13.0%	21.6%(11)	4.1%(2)		
**Disease-related**					
Time on dialysis (months)	20.0 (12–36)	20.5(11–36)	20(12–36)	0.83	
Time post transplant (months)	72.3 ±42.4	70.4 ±44.5	74.3 ±40.5	0.61	
Pre transplant treatment modality					
Dialysis	87.0%	88.2%(45)	85.7%(42)	0.46	0.80(0.24–2.57)
Pre-dialysis	13.0%	11.8% (6)	14.3% (7)		
**Therapy-related**					
Number of drugs per day	12.7±4.3	6.9 ± 2.3	6.7 ± 2.5	0.63	
Number of dosage times per day					
Two times		100.0%	98.0%(50)	0.32	
Three times		0	2.0% (1)		
Donor					
Living	97.0%	98.0%(50)	95.8%(47)	0.53	0.47(0.041–5.35)
Deceased	3%	2.0% (1)	4.1% (2)		
**Micro level**					
Good acceptability of transplant team	99%	98% (50)	100% (49)	0.24	
**Meso level**					
City of origin					
Same city of transplant center	29%	27.5%(14)	30.6%(15)	0.44	1.16(0.49─ 2.76)
Others	71%	72.5%(37)	69.4%(34)		
Distance to transplant center					
Absolute (miles)	45.5 (0–96)	66.0(0–96)	43.0(0–96)	0.46	
Till 62 miles	54%	47.1%(24)	61.2%(30)	0.15	1.77(0.80**─** 3.93)
More than 62 miles	46%	52.9% (27)	38.8%(19)		
**Macro level**					
No private health insurance	27%	35.3%(18)	18.4%(9)	0.04	2.42(0.96**─** 6.10)

The T-Test, Chi-square, Mann-Whitney, or Fisher´s tests were used to compare adherent with non-adherent. Normality test checked by Kolmogorov-Smirnov and Shapiro-Wilk tests.

*Reference wages in Brazil in 2010 –US $ 278.7.

In multivariate analysis, only having a family income higher than five reference wages was independently associated with NA outcome (OR 5.0,1.01–25.14, p = 0.04). Table 4.

**Table 4:** Multivariable Logistic Regression Of Correlates Of NA.

Variable	OR (95% IC)	p-value
Distance > 100 km/62 miles	1.7 (0.73–3.8)	0.22
Family income ≥ a 5 salaries	5.0 (1.01–25.14)	0.04
Private health insurance	1.7 (0.6–4.6)	0.28

Adjusted for variables that had P < 0.20 in univariate analysis: family income; distance to transplant center and private health insurance.

## Discussion

Based on the triangulation of assessment methods for NA, this cross-sectional study involving Brazilian KTx patients, revealed that 51% of subjects were non-adherent to their immunosuppressive medications. This peculiar population is provided with free immunosuppressants and with a more favorable access to health care after transplantation, especially those patients with only public health insurance before KTx. Our study is one of the few that have assessed the multilevel correlates of NA in transplantation. It is the first, to our knowledge, to study the correlates of NA in Brazilian KTx patients. Through multivariate analyses, we found that patients with higher incomes showed more NA, an unexpected yet reasonable finding from the gift-exchange relationship [25]. One hypothesis is that underprivileged patients who are provided with limited care through the public health system before transplantation, experience a double gift after KTx because of the provision of free immunosuppressives and a more structured health assistance program after transplantation. Those already covered by private insurance (i.e. higher income levels) might not perceive the gift as such, resulting in a more careless intake of immunosuppressants.

The high level of NA (51%) in our study can only be compared to a few other Brazilians reports [22,26,27,28]. Two of them assessed NA only after graft loss, referring to only the top of the iceberg and therefore not a valuable resource for comparisons [26,27]. Brahm et al reported a 58.7% prevalence of NA in KTx patients using pharmacy refill records [28]. Our group, in the validation study of the self-report BAASIS, thus applying only one method, found that 34% of patients were non-adherent [22]. None of the reports used combined methods.

Considering the potential consequences, the frequency of NA of our study is somehow worrying. Overall, a median of 22% non-adherent recipients (varying from 1.4 to 66.7%) has been reported in cross-sectional studies [8]. On the other hand, if we only consider self-report as the diagnostic method, our prevalence was higher compared to other studies that describe rates ranging from 12 to 24% [21,29]. Our NA frequency could also result from the application of a more accurate methodology, the triangulation method. Although it was not fully evaluated, since it was not an objective of our study, the prevalence of NA increased with the use of associated methods of diagnosis.

Based on the methodology of triangulation, applying three measurements tools, a unique study involving KTx patients found a similar percentage (60%) of NA [21]. Compared to our prevalence of 48%, a study by Schmiid-Mohler et al. involving KTx recipients in Switzerland described a lower frequency of 26.4% using a composite score of two methods (BAASIS and collateral report) [30].

However, our rates of NA can still be considered elevated, because immunosuppressive medications are freely provided to transplant patients in Brazil. In other countries with the same health policy of free access to immunosuppressives, the prevalence of NA was lower and varied from 34 to 45%, based on only one method of NA assessment [31,32]. This finding highlights the complexity of NA behavior and reveals the need for further exploration of the reasons for this high prevalence.

We investigated many potential correlates, comprising the four levels of NA based on the Ecological Model. Some classic correlates associated with NA were not detected in our study, such as younger age and male sex) [26, 33]. NA was associated with a higher socioeconomic level, characterized by the additional support provided by private health insurance and a better family income. This subgroup of ´privileged people´ would theoretically have a lower risk of NA since they have easier access to information and treatment. In contrast to our findings, the behavior is expected in people with a lower socioeconomic profile [34]. One could speculate that the supposed easy availability of the transplant option as a treatment could influence reckless behavior in the care of their kidney graft by that privileged individual.

Our study has some important limitations. First, the convenience sample was relatively small and composed mostly of recipients of living donor grafts from a single center. Although the deceased donors’ recipient numbers are not representative of our study, recent substantial evidence favors living kidney allograft as a risk factor for NA in KTx [35]. Consisting of young Caucasian individuals, between 40–45 years who mostly received living grafts, our sample reflects the actual demographic patterns of the Brazilian transplanted population [36]. In a large country like Brazil, future studies representing the Brazilian transplant population will be necessary to determine the actual prevalence of NA. Besides we opted to perform a study involving potential correlates of NA comprising the four levels of ecological framework, additional factors could be studied. We believe individual-related variables such as psychosocial variables (e.g. self-efficacy, self concept, and mental distress) and health system-related patterns (multidisciplinary care, specific skills of health team) should be further dissected. Future research should include larger samples to have more power to study multiple factors of NA, preferably involving several centers. Nevertheless, our results bring relevant epidemiology insights in the absence of previous studies among the Brazilian population, as well a very few studies involving the specific framework of the full immunosuppressant access system. Our results also reinforce the need of research involving the multi levels of health care, mainly at macro level involving the organization of health care policies [37].

## Conclusions

Using appropriate methodology of triangulation, we studied prevalence and correlates of NA immunosuppressives in a population of Brazilian post adult KTx patients, who received full-access to immunosuppressives. High levels of NA were found and having a high income was the single variable associated with NA. These findings are unexpected for a population from a developing country, like Brazil, where the underprivileged conditions are more likely to be associated with NA behavior. They also reinforce the need for the evaluation of the correlates to NA in specific populations before designing strategies to reduce NA.

## Supporting Information

S1 Appendix Database of all collected information. (SAV)
